# Refractory macular hole repaired by autologous retinal graft and blood clot

**DOI:** 10.1186/s12886-018-0898-8

**Published:** 2018-08-29

**Authors:** An-Lun Wu, Lan-Hsin Chuang, Nan-Kai Wang, Kuan-Jen Chen, Laura Liu, Ling Yeung, Tun-Lu Chen, Yih-Shiou Hwang, Wei-Chi Wu, Chi-Chun Lai

**Affiliations:** 1 Department of Ophthalmology, Chang Gung Memorial Hospital, No.5, Fu-Hsin Rd., Fuxing St., Guishan Dist, Taoyuan, 33375 Taiwan; 2 College of Medicine, Chang Gung University, No.259, Wenhua 1st Rd., Guishan Dist, Taoyuan, 333 Taiwan; 3 Department of Ophthalmology, Chang Gung Memorial Hospital, No.222, Maijin Rd., Anle Dist, Keelung, 204 Taiwan; 4 Edward S. Harkness Eye Institute, Department of Ophthalmology, Columbia University, 635 west, 165th street, New York, NY 10032 USA

**Keywords:** Refractory macular hole, Retinal graft, Autologous blood clot

## Abstract

**Background:**

To evaluate the surgical technique using autologous retinal graft (ARG) and autologous blood clot (ABC) for the management of refractory macular holes (MHs).

**Methods:**

This study was a retrospective, consecutive, interventional case series. Six eyes of 6 patients who underwent vitrectomy combined with ARG and ABC for the treatment of refractory MH were reviewed. Visual and anatomic outcomes were evaluated.

**Results:**

The mean age was 59.0 ± 9.9 years. All cases had multiple vitreoretinal procedures including vitrectomy and gas fluid exchange before patient presentation. The average numbers of vitrectomies were 2.3 ± 0.5, and those of gas fluid exchange were 3 ± 1.7. Closure of the macular hole was achieved in four (66.7%) cases at last follow-up. The mean follow-up time was 25.2 ± 15.6 months. The averaged BCVA before and after 12 months of the surgery improved from 20/591 to 20/244.

**Conclusions:**

This surgical technique using ARG and ABC provide an option for the treatment of refractory MHs.

**Electronic supplementary material:**

The online version of this article (10.1186/s12886-018-0898-8) contains supplementary material, which is available to authorized users.

## Background

Pars plana vitrectomy combined with internal limiting membrane (ILM) peeling and gas tamponade is considered a standard approach for treating macular holes (MHs) [1]. The inverted ILM flap technique had been reported to improve the success rate in difficult cases [2]. The anatomic closure rate in a single operation could reach nearly 90% or higher [3, 4]. Repeat fluid-gas exchange closed most of the open holes after primary vitrectomy with ILM peeling [5]. However, surgical failure is still reported despite these high success rates.

Various surgical strategies have been introduced as adjunctive procedures to attempt closure of the refractory MH; these include enlargement of the previous ILM peel [6], use of heavy silicone oil for tamponade [7], pedicle ILM flap technique [8], lens capsular flap transplantation [9], autologous free ILM flap [10–12], and autologous neurosensory retinal free flap [13].

Autologous neurosensory retinal free flap transplantation was first proposed by Grewal et al. [13], is a surgical technique used when no ILM was available to repair the MH. And another case report was presented to show the feasibility of using this method [14]. Although anatomical success and functional improvement could be reached, longer follow-up and larger sample sizes are needed for further study. Also, the risk of graft dislocation both intraoperatively and postoperatively remain an issue. Our group reported a surgical approach using the addition of autologous blood clot (ABC) appear to improve the limitation [15]. Therefore, we combined the autologous retinal graft (ARG) and ABC technique together as a macular plug to increase stability and keep the graft in place. The objective of this study was to present the efficacy of this technique in treating refractory MHs after failed surgeries with ILM removal or transplantation.

## Methods

This study was a retrospective, consecutive, interventional case series. This ARG and ABC technique was conducted in six patients with refractory MH by 23-gauge vitrectomy. All surgeries were performed by one of the authors (C.-C.L.) at the Department of Ophthalmology, Chang Gung Memorial Hospital. This study adhered to the guidelines of the Declaration of Helsinki and was approved by the Institutional Review Board at Chung-Gang Memorial Hospital, Taiwan.

The inclusion criteria were as follows: (1) clinical presentation of an unclosed MH after previously receiving vitrectomy and ILM removal; (2) no remaining ILM within the vascular arcade in the posterior pole and treatment via combined pars plana vitrectomy with ARG and ABC; and (3) a follow-up period of more than 1 year after the vitrectomy with ARG and ABC. Patient records were reviewed, and the following data were collected: age, gender, past ocular history, preoperative best-corrected visual acuity (BCVA), and preoperative optical coherence tomography (OCT). Postoperative assessments were planned at 1 day, 1 week, 1 month, 3 months, 6 months, and 12 months after surgery; then, patients were followed up every 6 months. Extra visits may occur when there is a clinical need. BCVA and OCT were evaluated at each follow-up visit, except on the first postoperative day in which the fundus was prohibited from being viewed clearly. BCVA using a Snellen chart was converted to the logarithm of minimum angle of resolution (logMAR) for analysis purposes.

**Fig. 1:**
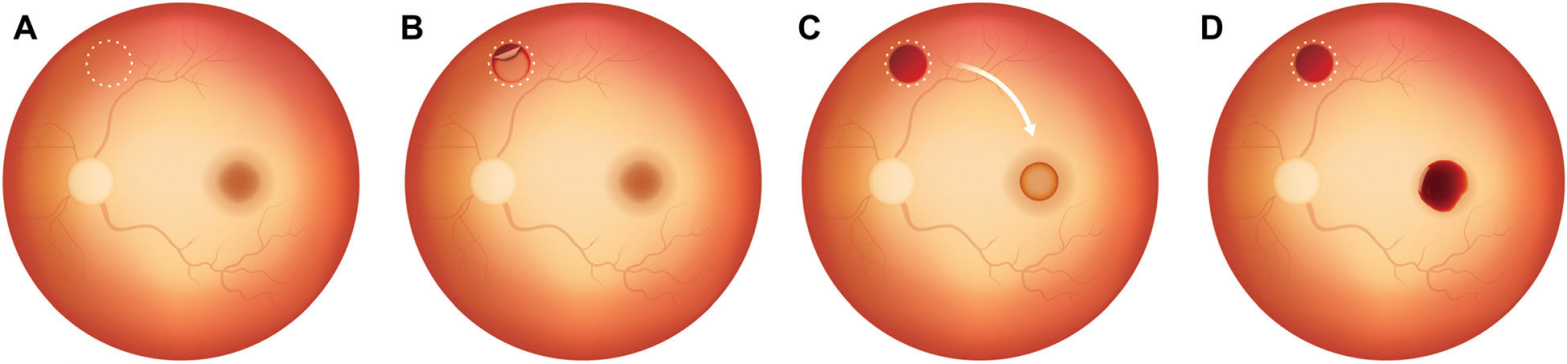
Schematic drawings showing autologous retinal graft transposition with autologous blood clot surgical technique for repairing refractory macular holes. (**a**) Select the retinal graft harvest site outside the vascular arcade. White dotted circle indicates the area of an autologous retinal graft that is facilitated by laser photocoagulation. (**b**) The edge of the retinal graft was cut using vertical scissors. (**c**) Retinal graft was obtained and gently moved toward insertion within the macular hole. (**d**) Retinal graft was stabilized by placing autologous fresh blood over it. The fresh blood soon became a clot on the surface of the macula, and the retina graft and blood clots to seal the hole in a few minutes as a macular plug


Diagrammatic representation of surgical procedure is outlined in Fig. 1. All cases underwent standard 23-gauge three-port, transconjunctival, sutureless microincision vitrectomy (Constellation; Alcon, Fort Worth, TX) with retrobulbar anesthesia. After the indocyanine green (ICG) staining procedure (concentration: 0.125 mg/ml in 5% glucose water) to make sure there was no remaining ILM within the vascular arcade in the posterior pole, excessive ICG was immediately removed via suction. The ARG harvest site was selected outside the vascular arcade and facilitated by laser photocoagulation in a circular manner. To achieve an effective laser burn, full thickness retinal burns were created at different power settings, from 200 mW to 300 mW and at different pulse durations, from 0.2 ms to 0.3 ms. The size of the graft was intended to be the same as the size of the MH and was harvested using scissors. Then, the retinal graft was gently moved to be inserted into the MH. Graft dislocation was prevented by lowering the intraocular pressure setting to reduce turbulence in the fluid stream. Further, the position of the graft proper was secured by trapping the edge of the graft under the edge of the hole. After the graft was manipulated into a proper position inside the hole, approximately 1 mL of fresh blood obtained from the patient’s antecubital vein was collected into a syringe, with strict attention to aseptic precautions after disinfecting the entry site, and allowing it to dry completely as described before [15]. One to two drops of the fresh blood were then injected gently to cover the MH, using a back-flush needle mounted on a 1-mL syringe. The fresh blood soon became a clot on the surface of the macula, and the retina graft and blood clot sealed the hole in a few minutes as a macular plug. Afterwards, fluid–air exchange was performed. If the flap was disturbed during the gas-fluid exchange, more blood could be applied. At the end of the operation, the air was replaced with 20% sulfur hexafluoride gas. Patients were asked to remain face down for one week after surgery. The key steps of this technique are outlined in the Additional file 1.

Additional file 1:Video that illustrates key steps of autologous retinal graft and blood clot technique for the management of refractory macular holes. (MP4 17160 kb)


## Results

A total of six cases (six eyes in six patients: four female and two male) with refractory MH underwent surgery using the “ARG and ABC” technique. Characteristics of the patients and surgical results are summarized in Tables 1 and 2 separately. Mean age at the time of the surgery was 59.0 ± 9.9 years. The mean basal MH size before autologous transposition of the graft was 978.5 ± 441.0 μm. The mean minimum opening of MH was 538.0 ± 184.9 μm. In failed cases, the mean basal MH size was 1047.5 ± 200.5 μm, and the mean minimum opening of MH was 697.0 ± 88.0 μm before surgery. Regarding the pathologies of MH in our study, 4 eyes (66.7%) were classified as idiopathic MH. And one MH occurred after successful rhegmatogenous retinal detachment surgery. The last case of MH had coexisting history of branch retinal vein occlusion with sectoral photocoagulation. All patients were pseudophakic at the beginning of the study. All cases had multiple vitreoretinal procedures including vitrectomy and gas fluid exchange before patient presentation. The average number of vitrectomies before ARG was 2.3 ± 0.5, and the average number of gas fluid exchanges was 3 ± 1.7. Closure of the MH was achieved in four out of the six (66.7%) patients after ARG in the study.

**Table 1:** Characteristics of patients undergoing autologous retinal graft and blood clot for refractory macular holes

Patient no.	Age (years)	Sex	Eye	Minimal diameter of MH (μm)	Basal MH size (μm)	MH status after ARG + ABC	Preoperative Lens status	Preoperative BCVA	Postoperative BCVA at 1 year	Follow-up
1	56	F	OS	219	576	Closed	Pseudophakic	20/1000	20/100	51
2	41	M	OS	657	832	Closed	Pseudophakic	20/400	20/200	36
3	61	M	OD	389	548	Closed	Pseudophakic	20/333	20/67	24
4	67	F	OS	569	1820	Closed	Pseudophakic	20/400	20/200	15
5	68	F	OS	785	1248	Open	Pseudophakic	20/400	20/400	13
6	61	F	OS	609	847	Open	Pseudophakic	20/2000	20/2000	12

*ARG* = autologous retinal graft, *ABC* = autologous blood clot, *BCVA* = best-corrected visual acuity, *MH* = macular hole

**Table 2:** Demographics and surgical results among all cases (*n* = 6)

Factor	
Male/Female (no.)	2/4
Age (yrs)	59.0 ± 9.9
Idiopathic MH, no. (%)	4 (66.7)
Axial length (mm)	25.63 ± 1.76
Follow-up (months)	25.2 ± 15.6
Basal MH size (μm)	978.5 ± 441.0
Minimum opening of MH (μm)	538.0 ± 184.9
Basal MH size in failed cases (μm)	1047.5 ± 200.5
Minimum opening of MH in failed cases (μm)	697.0 ± 88.0
Preoperative BCVA (logMAR)	20/591 (1.47 ± 0.31)
Postoperative BCVA (logMAR)	
1 mo	20/691 (1.54 ± 0.41)
3 mos	20/342 (1.23 ± 0.27)
6 mos	20/310 (1.19 ± 0.44)
12 mos	20/244 (1.09 ± 0.52)
BCVA at last visit, no. (%)	
Improved	4 (66.7)
No change	2 (33.3)
Worse	0 (0)
MH closed, no. (%)	4 (66.7)

*BCVA* = best-corrected visual acuity, *logMAR* = logarithm of the minimum angle of resolution, *MH* = macular hole

**Fig. 2:**
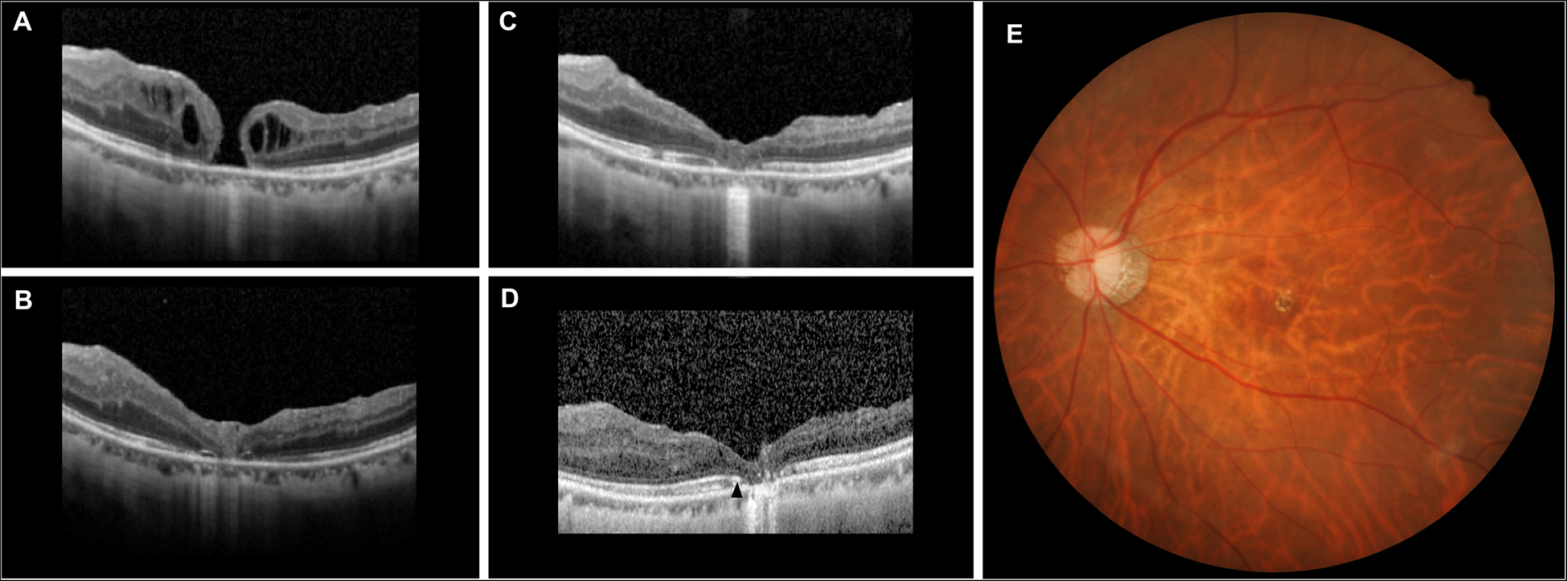
Results of autologous retinal graft and blood clot in a 55-year-old refractory macular hole patient. Despite 4 pars plana vitrectomies with ILM peeling and additional 5 gas-fluid exchanges, spectral domain optical coherence tomography images (SD-OCT) showed the hole persisting (**a**). The base diameter of the MH before the retinal graft transplantation was 576 μm, and the minimum opening of the MH size was 219 μm. After the procedure, the macular hole was successfully closed at 3 months (**b**) and 12 months (**c**). The fovea remained stable and the patient’s visual acuity improved from 20/1000 before the surgery to 20/100 at the final visit. SD-OCT imaging (**d**) and fundus photograph (**e**) obtained at 51 months postoperatively. SD-OCT showing partial restoration of external limiting membrane and gradually reconstructed ellipsoid zone (black arrowhead)

Intraoperatively, all eyes had successful transposition of the ARG and no intraoperative complications related to this technique were noted. The mean follow-up time was 25.2 ± 15.6 months. The averaged BCVA in logMAR before and after 12 months of the surgery were 20/591 (1.47 ± 0.31 logMAR) and 20/244 (1.09 ± 0.52 logMAR), respectively. Overall, 4/6 eyes with a closed hole after ARG gained visual acuity improvement, 2/6 eyes without a closed hole remained stable, and no eyes experienced a deterioration of the best corrected visual acuity after ARG surgery. The blood clot could be observed approximately one week after surgery. No postoperative complications developed during the follow up period. In the follow-up OCT, the graft appeared dislodged after the operation and no graft tissue was visible in the two eyes without closed holes. A reduced ellipsoid zone (EZ) line gap with partial restoration of the external limiting membrane and EZ was noted in one eye at last follow up regarding the recovery of the fovea microstructure post operatively 51 months later (Fig. 2).

## Discussion

The treatment of an idiopathic macular hole could achieve a 90% closure rate or more with a modern vitrectomy [3, 4]. Some MH patients, such as those with a chronic large hole, high myopia, or trauma may require multiple surgeries. Treatment of refractory MH became a challenging issue for macular hole closure. Surgical management using ARG with ABC demonstrated the efficacy of a surgical option in refractory MHs.

The described technique may be used in patients with refractory MHs who underwent multiple surgeries and who had no remnant ILM. However, the closure rate of retinal graft transplantation for refractory MHs compares less favorably with those using ILM free flap transplantation [10–12]. This might be partly because anatomic success is more difficult to achieve in refractory MHs with repeated surgeries and when ILMs had been removed. Functional recovery after surgery is gradual and partial restoration of the external limiting membrane and EZ could continue suggesting that the retinal graft placed inside a macular hole may form a bridge to repair the fovea laminar structure from the graft edges. However, further studies are certainly needed to investigate if ARG could not only serve as a scaffold but also retain some function and further promote the reparation of the outer retinal layers.

Surgical approaches for refractory MH are limited. Improved outcomes have been reported with autologous ILM flaps [10–12]. However, harvesting a new suitable ILM free flap can be problematic in eyes with previous extensive ILM peeling. Lens capsular flap transplantation may be a solution for refractory MH when the ILM has already been removed [9]. However, the technique is not appropriate in phakic eyes if concomitant cataract surgery is not indicated and cannot be used in pseudophakic eyes with an open posterior capsule. Grewal and Mahmoud described an alternative approach involving the use of an autologous neurosensory retinal free flap for closure of refractory MH [13]. The differences between their procedure and ours are in considering the size of the transplanted flap and its placement. In our surgical procedure, we placed a similarly sized retinal graft inside the macular hole instead of a free larger flap covering on it. We consider that persistent MHs close more efficiently and promptly when graft tissue becomes a filler, because the tissue may serve as a scaffold and a stronger bridge for glial cell proliferation. More importantly, we hypothesized to use ABC not only as a glue to keep the subsequently placed graft in place and reduce the risk of graft dislocation after the surgery, but also as a potential autologous adjuvant to augmenting the healing processes [15]. Additionally, the mixture of ARG and ABC formed a macular plug that sealed the MH shortly after application. This may maintain the dryness of the MH, prevent vitreous fluid from moving into the defect, and thereby ensure that the glial proliferation and migration closes the hole completely theoretically. However, further study is needed to arrive at a definitive conclusion of the mechanism.

The most difficult part of the transposition of the retinal graft technique is the transferring procedure from the harvest site to precisely insert the graft inside the macular hole. Grewal and Mahmoud developed the Perfluoro-n-octane heavy liquid (PFC) (Perfluoron; Alcon) assisted neurosensory retinal free flap transposition technique in their case [13]. The tamponade effect from the PFC could prevent the graft from floating away on intraocular currents during surgery before direct PFC–silicone oil exchange. In contrast to their method, we found that the ARG tissue could be mechanically tucked and positioned inside the MH until completion of the autologous blood clot. However, the most important point during this procedure is to avoid potential iatrogenic trauma to the fovea tissue.

In this series, the aspiration of fluid was slowly performed via fluid-air exchange, and we did not perform further fluid aspiration after a 5 to 10-min wait after the initial fluid-air exchange to maximize vitreous cavity dehydration [16, 17]. Further, there is no need to aspirate the last drop of vitreous cavity fluid in avoidance of disturbing to the macular plug; this is because the hole is, theoretically, temporarily sealed after blood clot formation. And further gas tamponade is important to maintaining the dryness of the macula, which prevents the vitreous fluid from moving into the hole, and ensures that the retinal graft can be placed inside the MH as a bridge to help the repair process.

Graft dislocation was detected by OCT one month postoperatively in our two failure cases. The patients declined to receive any further treatment, however, the visual acuity revealed no deterioration after the surgery. We noted that these two cases were idiopathic MHs and the hole size was larger than the other four cases preoperatively. Given that hole and graft size cannot be accurately measured intraoperatively, the graft prepared may not have been large enough to fill the whole MH. Additionally, a shrinkage of the graft tissue may happen when cutting a graft, or contracture may occur during the healing process. In this situation, it was difficult to secure the graft tissue under the hole margin, causing graft dislodgement during the postoperative period. Regarding the toxicity issues due to blood products and fibrin degradation products used in this technique [18], although blood may leak into the subretinal space and cause damage to the photoreceptors, safety concerns are reduced because the graft tissue barrier serves as a filler at the hole. Although this study contains the postoperative visual acuity, further study measuring electroretinogram and microperimetry may provide more information regarding the functional outcomes.

## Conclusion

Management of refractory MH presents a surgical challenge. ARG and ABC technique may be used in cases of refractory MH who have undergone multiple surgeries, and where no remnant ILM is present within the arcade vessels. Also, this surgical technique had a positive effect on visual function in all of the closure cases. Further study in a larger population that directly compares this technique with other techniques is warranted.

## Abbreviations

ABC: Autologous blood clot
ARG: Autologous retinal graft
BCVA: Best-corrected visual acuity
EZ: Ellipsoid zone
ICG: Indocyanine green
ILM: Internal limiting membrane
logMAR: Logarithm of minimum angle of resolution
MH: Macular hole
OCT: Optical coherence tomography
PFC: Perfluoro-n-octane heavy liquid
